# Stress memory responses and seed priming correlate with drought tolerance in plants: an overview

**DOI:** 10.1007/s00425-022-03828-z

**Published:** 2022-01-23

**Authors:** Xun Liu, Wenli Quan, Dorothea Bartels

**Affiliations:** 1 Institute of Molecular Physiology and Biotechnology of Plants (IMBIO), University of Bonn, Kirschallee 1, 53115 Bonn, Germany; 2 College of Bioengineering, Sichuan University of Science & Engineering, Zigong, 643000 China; 3 Key Laboratory for Quality Control of Characteristic Fruits and Vegetables of Hubei Province, College of Life Science and Technology, Hubei Engineering University, Xiaogan, 432000 Hubei China

**Keywords:** Cross-stress tolerance, Dehydration stress, Drought priming, Drought tolerance, Epigenetic perspective, Plant stress memory, Seed priming

## Abstract

**Main conclusion:**

**Environmental-friendly techniques based on plant stress memory, cross-stress tolerance, and seed priming help sustainable agriculture by mitigating negative effects of dehydration stress.**

**Abstract:**

The frequently uneven rainfall distribution caused by global warming will lead to more irregular and multiple abiotic stresses, such as heat stress, dehydration stress, cold stress or the combination of these stresses. Dehydration stress is one of the major environmental factors affecting the survival rate and productivity of plants. Hence, there is an urgent need to develop improved resilient varieties. Presently, technologies based on plant stress memory, cross-stress tolerance and priming of seeds represent fruitful and promising areas of future research and applied agricultural science. In this review, we will provide an overview of plant drought stress memory from physiological, biochemical, molecular and epigenetic perspectives. Drought priming-induced cross-stress tolerance to cold and heat stress will be discussed and the application of seed priming will be illustrated for different species.

## Introduction

Dehydration stress is one of the major environmental factors affecting the survival rate and productivity of plants. Dehydration leads to low water availability and adversely affects food security (Abdelraheem et al. 2019; Mahmood et al. 2020). In plants, the amount of water loss by transpiration exceeds the amount of water up-take via roots, giving rise to dehydration stress (Chawla 2019). Water deficit causes cellular dehydration, accumulation of reactive oxygen species (ROS), cell death, and ultimately affects metabolism and growth (Farooq et al. 2009). To overcome this unfavorable condition, dehydration-tolerant crops have been developed through breeding programmes or transgenic technologies or genome modification approaches (Mahmood et al. 2020). More recently, potential solutions based on plant immune systems, including plant stress memory, cross-stress tolerance, and seed priming have emerged as efficient and favorable approaches for enhancing plant tolerance and crop yield without employing genetic engineering technologies (Wojtyla et al. 2020). Therefore, a better understanding of the mechanisms of these approaches is important for biotechnological innovation of plant resilience.

Due to frequent climatic changes and extreme conditions occurring recently plants are likely to be exposed to multiple abiotic stresses during their whole life span, instead of single stress events (Li and Liu 2016). To survive stress and to adapt to harsh environments, plants have to find suitable ways to respond to recurrent stresses. It has been observed that pre-exposure to a mild biotic or abiotic stress can prepare plants for subsequent severe stress exposures (Walter et al. 2011; Ramírez et al. 2015). This phenomenon is referred to as “plant priming”, which is considered as a potential way to improve stress tolerance, and it is related to “plant stress memory” (Bruce et al. 2007). The expression “plant priming” is generally used in the context of biotic stresses and application of chemicals for the first exposure, while the similar process is termed “hardening” or “acclimation” in the context of abiotic stress (Sinclair and Roberts 2005; Chen et al. 2012; Hilker et al. 2016; Savvides et al. 2016). The concept of stress memory represents an intrinsic response to repeated stress events (Avramova 2015). Many efforts have been made to explore the mechanisms of stress memory in different plant species which have encountered diverse stresses (Ramírez et al. 2015; Walter et al. 2011; Wang et al. 2014, 2015; Shukla et al. 2015; Sun et al. 2018). The results show that stress memory is involved in modifications at different levels, including morphological, physiological, transcriptional, translational, and epigenetic levels (Kinoshita and Seki 2014; Sun et al. 2018).

Cross-stress tolerance represents the tolerance to a second strong stress which differs from the first stress after experiencing a primary mild stress. Cross-stress tolerance is achieved by the activation of multiple stress signaling pathways during the first stress encounter, and these activated pathways work synergistically or antagonistically during subsequent stress events (Hossain et al. 2018). It has been reported that the cross-stress tolerance gained from a single stressor can lead to the tolerance of multiple stresses (Herms and Mattson 1992; Li and Gong 2011; Ferreira-Silva et al. 2011; Zhang et al. 2013; Li et al. 2014; Faralli et al. 2015). Cross-stress tolerance provides the possibility to understand common signaling molecules and to compare individual responses during different stresses (Hossain et al. 2016).

In addition to priming on the whole plant level, memory and cross-stress tolerance, the priming of seeds is also pivotal in managing stressful conditions. Seed priming is a treatment applied before sowing during seed imbibition (Sen and Puthur 2020). Seed imbibition involves three stages of seed development: rapid water uptake, saturation of water uptake, and water uptake together with the onset of cell division and growth. The second stage appears to be the critical stage for seed priming (Cheng et al. 2017). Seed imbibition is a complex physiological and biochemical process, and many metabolic processes take place during the second stage, including restoring of mitochondrial and cellular integrity, mobilizing of stored energy, synthesis of RNAs and proteins (He and Yang 2013). Seed priming is a crucial technology for uniformity of germination and seedling establishment under adverse environmental conditions.

This review summarizes the physiochemical and molecular perspectives of plant stress memory, and illustrates how cross-stress tolerance and seed priming can contribute to stress tolerance.

## Physiological and biochemical perspectives of drought stress memory

To optimize growth and reproduction in frequently changing environments, plants may adjust their physiology to give rise to structural and physiological adaptations (Fleta-Soriano and Munné-Bosch 2016). Many plant species display a drought stress memory on the physiological and biochemical level, to minimize water loss, to obtain ROS homeostasis, alterations of photosynthetic rates, variations of phytohormone contents, or changes in biomass (Ding et al. 2014; Ramírez et al. 2015; Wang et al. 2015; Li et al. 2016; Abdallah et al. 2017; Neves et al. 2017).

The rate of water loss from plant leaves has been proposed as a basic parameter to reflect the growth conditions of plants. In *Arabidopsis thaliana* plants, which underwent a drought stress treatment, the stomata were still partially closed during the well-watered recovery period, which is beneficial for water conservation if exposed to a subsequent dehydration stress (Virlouvet and Fromm 2015). A repetitive dehydration/rehydration system was developed by Ding et al. (2012) to determine whether *A. thaliana* plants retain a drought stress memory. A significant lower water loss rate was observed during the second, third and fourth dehydration stress compared to the first stress (Ding et al. 2012). Similarly to *A. thaliana* (Ding et al. 2012), maize or the desiccation tolerant resurrection plant *Craterostigma plantagineum* plants pre-exposed to dehydration had a higher relative leaf water content (RWC) than the non-trained plants when exposed to a subsequent dehydration episode (Ding et al. 2014; Liu et al. 2019). Studies on three contrasting potato genotypes (*Solanum tuberosum* L.) found that a pre-treatment of drought acclimation cycles reduces leaf wilting, induces thicker cuticular layers and more open stomata under a subsequent drought stress, compared to a direct application of drought without pretreatment (Banik et al. 2016).

Generally, ROS are kept at relatively low levels in plants under optimal growth conditions with a balance between ROS scavenging and ROS production (Hossain et al. 2016). Elevated ROS production is caused by stress, and the major source of ROS production are organelles with high oxidative activities (eg. peroxisomes, chloroplasts, and mitochondria) (Sharma et al. 2012). Studies on a drought sensitive cultivar of olive cv. *Chétoui* showed that pre-exposure to drought induces a better maintenance of ROS homeostasis by increasing contents of polyphenols and activities of ROS scavenging enzymes like guaiacol peroxidase (GP), superoxide dismutase (SOD) and catalase (CAT) decreasing the content of hydrogen peroxide (H_2_O_2_) and malondialdehyde (MDA) (Abdallah et al. 2017). Amoah et al. (2019) reported that lipid peroxidation, reactive oxygen species, membrane stability, antioxidant enzyme activities, and the contents of H_2_O_2_ and osmolytes increased in wheat plants with drought acclimation under subsequent water stress treatments compared to non-acclimated plants.

The photosynthetic rate is sensitive to abiotic stress, especially dehydration stress. Decrease of photosynthesis under water deficit is initially caused by low levels of CO_2_ diffusion from the atmosphere to the carboxylation site (Lawlor and Tezara 2009; Wang et al. 2015). Pre-exposure to mild drought could maintain the photosynthetic electron transport in the photosynthetic apparatus of barley plants during a subsequent cold stress (Li et al. 2016). A better photosynthesis rate during severe stress was obtained by exposing winter wheat plants to a mild drought treatment (Li et al. 2014). A drought stress memory was also reported for *Aptenia cordifolia* plants which encountered repeated stresses (Fleta-Soriano et al. 2015). The *A. cordifolia* plants exposed twice to drought stress had increased chlorophyll a/b ratios compared with a reference group not exposed to a mild stress (Fleta-Soriano et al. 2015).

Abscisic acid (ABA), an essential phytohormone, increases in response to dehydration through a complex equilibrium of synthesis, degradation or conjugation (Kim 2012; Finkelstein 2013). A study of *A. cordifolia* plants found that ABA levels increased in leaves which encountered two subsequent drought stress episodes compared to plants which were only exposed once (Fleta-Soriano et al. 2015). Neves et al. (2017) observed that citrus plants which underwent multiple exposures to dehydration also had higher ABA levels compared to plants stressed only once. Research on spring wheat (*Triticum aestivum* L. cv. Vinjett) showed that the wheat plants pre-exposed to a moderate water deficit had higher concentrations of ABA compared to non-primed plants, and the pre-exposure eventually resulted in higher grain yields (Wang et al. 2015).

In plants, a drought stress memory can positively influence biomass or grain yield through efficient regulations of water loss, ROS levels, and photosynthesis. In long-term stress memory experiments, the yield of potatoes was investigated using primed or non-primed tubers (Ramírez et al. 2015). Potato tubers which had been subjected to priming through a mild dehydration treatment had a higher tuber yield than the ones produced in well watered conditions, when the potatoes had been grown in similar conditions (Ramírez et al. 2015). The biomass of *Arrhenatherum elatius* plants which had been pre-exposed twice to drought stress was higher than that of plants which only encountered a single drought stress (Walter et al. 2011). Wheat plants were used to explore the possible effect of drought stress memory during plant development (Wang et al. 2014). The ascorbate peroxidase activity and photosynthesis rate of wheat plants primed before anthesis were higher whereas the content of malondialdehyde was lower than in the non-primed wheat plants, and higher grain yield was obtained than in non-primed plants during a severe drought encountered during the grain filling stage (Wang et al. 2014). A moderate drought stress during the vegetative state in spring wheat alleviated yield loss caused by drought during the grain filling stage (Wang et al. 2015).

## Molecular mechanisms and epigenetic changes in drought stress memory

Research on drought stress memory suggests that regulatory mechanisms on the transcriptional level differ in response to a single stress stimulation and repeated stress stimulations (Avramova 2015; Berry and Dean 2015). Changes of gene expression patterns related to stress memory are often correlated with changes of the chromatin status (Campos and Reinberg 2009). The molecular responses of the stress memory act on two levels *cis*-mechanisms and *trans*-mechanisms. This means that a memory is generated on chromatin marks (including DNA methylations and histone modifications) and a memory is maintained by feedback loops and cytosol partitioning (Bonasio et al. 2010; Berry and Dean 2015; de Freitas Guedes et al. 2019).

Epigenetic mechanisms like DNA methylations, histone modifications and chromatin structure alterations play an important role in the regulation of gene expression which contribute to epigenetic inheritance in plants (Chinnusamy and Zhu 2009; Friedrich et al. 2019). Changes on the epigenetic level are often inherited or transmitted to the next generation through mitotic cell divisions (Kinoshita and Seki 2014).

### DNA methylation

DNA methylation is a heritable epigenetic mark which is linked to transcriptional repression (Ueda and Seki 2020). DNA methylation is catalyzed by DNA methyltransferases on the fifth carbon of cytosine bases symmetrically (CG and CHG, H = A, T, or C) and asymmetrically (CHH) (Han and Wagner 2014; Vyse et al. 2020). DNA methylation is a reversible process and associates with plant development and environmental responses (Hu et al. 2013). By comparing the DNA methylation patterns of drought sensitive and drought tolerant rice plants changes of DNA methylation were more frequent in drought tolerant rice plants than in drought susceptible rice plants under drought conditions (Wang et al. 2011). To investigate the role of genome-wide DNA methylation patterns in drought stress memory, seedlings of *A. thaliana* were used in a simulated drought treatment. However, the results showed no correlation between DNA methylation levels and gene expression patterns (Colaneri and Jones 2013). When on the other hand differential DNA methylation patterns were analyzed in rice plants using drought susceptible lines, drought tolerant lines, and their F1 hybrids DNA methylation was correlated with drought tolerance, and hypo-methylation of DNA was an indicator of drought tolerance (Joel 2013). DNA methylation patterns identified by DNA analysis provide evidence for drought-induced DNA methylation associated with acclimation responses in rice (Sapna et al. 2020). Genome-wide bisulphite sequencing of rice plants uncovered that dynamic and distinct patterns of differentially methylated DNA regions are related to drought stress memory. These methylated regions contribute to short-term repeated drought stresses by regulating activity of transposable elements and gene expression (Kou et al. 2021). Studies on wild strawberry (*Fragaria vesca*) indicated that repeated stress conditions lead to the acquisition of a stable epigenetic memory on the level of DNA methylation (De Kort et al. 2020). Recently, the whole-genome DNA methylation data of the resurrection plant *Boea hygrometrica* showed that DNA methylation has potential implications on dehydration stress memory (Sun et al. 2021).

### Histone modifications

Histone modifications, including histone methylation, acetylation, ubiquitination, phosphorylation, ADP-ribosylation, and sumoylation, take place in the N-terminal regions of histones through covalent modifications (Zentner and Henikoff 2013; Khan and Zinta 2016). Histone methylation predominantly occurs on lysine and arginine residues including mono-methylation, di-methylation, or tri-methylation (Bannister and Kouzarides 2011; Zentner and Henikoff 2013). The profiles of histone H3 tri-methylations of lysine 4 and lysine 27 (H3K4me3 and H3K27me3) have been studied for five dehydration stress memory genes in *A. thaliana* plants (Liu et al. 2014). The results revealed distinct memory responses and showed different activities of transcription during rehydration (Liu et al. 2014). H3K27me3 is a well-known chromatin repressor for developmentally regulated genes, but it did not block the transcription of dehydration stress-related genes (Liu et al. 2014). The histone modification profiles and the nucleosome occupancy of dehydration responsive genes (including *RD20*, *RD29A* and *galactinol synthase* (*GOLS2*)) changed during the transition from dehydration to rehydration in *A. thaliana* (Kim et al. 2012). The presence of RNA polymerase II increased during dehydration and decreased during rehydration, which correlated with transcript profiles (Kim et al. 2012). Correlation of active transcription with the alteration of H3K4me3 indicates that this chromatin mark participates in the transcription memory of these genes (Kim et al. 2012). Ding et al. (2012) reported that the relative high levels of phosphorylation of serine 5 (Ser5P) and H3K4me3 of RNA polymerase II persisted while the transcripts of trainable genes fall to a basal level during rehydration, which suggests a correlation with a drought stress memory. Histone acetylation is reversible by transferring the acetyl group to the side chains of lysine and it is able to neutralize the positive charge of the lysine residue (Khan and Zinta 2016; Vyse et al. 2020). The acetylation level of lysine 9 of H3K9ac is related to the active state of dehydration-induced gene expression (Kim et al. 2012). Genome-wide analysis of histone modifications in maize plants showed that drought stress-induced changes of histone modifications could persist after the stress relief. H3K4me3 correlated better with changes of gene expression than H3K27me3 and H3K9ac under drought stress (Forestan et al. 2020). The genome-wide chromatin landscape of five histone markers were determined in the moss *Physcomitrella patens* to describe its dynamics during development and drought stress (Widiez et al. 2014). Three activating histone marks (H3K4me3, H3K27Ac and H3K9Ac) exhibit significant changes during development and drought stress. The changes of histone marks and gene expression induced during drought stress are primed to persist during developmental transition (Widiez et al. 2014). Grafting rapeseed (*Brassica rapa* subsp. *oleifera*) onto turnip (*B. rapa* subsp. *rapa*) indicates that drought stress can be memorized and transmitted from turnip rootstock to rapeseed scion through the modification of histone H3K4me3 at the *Δ1-pyrroline-5-carboxylate synthetase 1–2* (*P5CS1-2*) gene (Luo et al. 2020).

### RNA molecules and alterations of chromatin structure

Chromatin changes can be achieved by exchanging canonical histone and specific histone variants, which is referred to as chromatin structure alteration or chromatin remodeling (Vyse et al. 2020). Replacement of histones with variants of different physical properties leads to epigenetic changes. Talbert and Henikoff (2014) proposed that exchange of different histone variants in the chromatin could be a mediating mechanism which takes place when plants encounter environmental changes. The chromatin structure can also be modified by RNA molecules (such as siRNAs, miRNAs, and long non-coding RNAs) through DNA modifications and recruitment of histone methyltransferases (Holoch and Moazed 2015). RNA-directed DNA methylation of cytosine is specific for CHH (H = A, T, or C) sequences (Singroha and Sharma 2019). Mozgova et al. (2019) reported that non-coding RNAs are also becoming important players of stress and stress memory responses.

In conclusion, chromatin modifications and gene expression are regulated at various levels during different phases of dehydration and rehydration. Evidence increases for epigenetic mechanisms of stress responses and memory in plants, but more studies are needed to understand the role of a drought stress memory in adaption of plants to dehydration.

## Cross-stress tolerance

Under natural field conditions, plants are likely to be exposed to different stresses at the same time or at different stages of their life cycle instead of being exposed to one stress with the same intensity as under experimental laboratory conditions. Thus, cross-stress tolerance is important for growth and development during the complete life cycle of a plant. Cross-stress tolerance may be obtained by establishing acclimation mechanisms, such as morphological changes, accumulation of specific transcription factors and protective metabolites, as well as epigenetic modifications (Munné-Bosch and Alegre 2013; Walter et al. 2013). Heat stress, freezing stress and drought stress all of them will cause cellular dehydration and induce acclimation mechanisms, which are partly similar to each other (Beck et al. 2007). Therefore, it is highly possible that activated acclimation mechanisms caused by one type of stress can prevent damage from other stresses which occur later. This phenomenon is termed cross-stress tolerance (Walter et al. 2013).

All processes involved in cross-stress tolerance are regulated by a complex network covering the interaction of multiple external and internal factors, permitting plants to adapt to changing environments (Munné-Bosch and Alegre 2013). The current review highlights the drought stress memory-induced cross-stress tolerance in plants. Drought priming-induced cross-stress tolerance to cold and heat stress has been observed and reported across various species (Table 1).

**Table 1:** Examples of drought stress-cross-tolerance (cold and heat) in plants

Primary stressor	Species	Cross adaptation	Responsible factors	References
Drought	*Coffea* spp.	Cold	Higher SOD, APX, GR, CAT activities; higher expression level of APXc, APXt + s, PX4; higher amount of non-enzyme antioxidants (TOC and ASC)	Ramalho et al. (2018)
Water stress + cold acclimation	Strawberry	Freezing tolerance	The expression of COR47 and COR78 orthologs	Rajashekar and Panda (2014)
Extreme drought	*Pinus nigra*	Cold hardiness	Higher soluble carbohydrates and chain length (ACL) of fatty acids	Kreyling et al. (2012
Drought priming	Wheat	Cold tolerance	Higher RWC and ABA content, higher GPX, SOD, APX and CAT activities; lower H_2_O_2_ content	Li et al. (2015)
Drought priming	Barley	Cold tolerance	Higher ABA and melatonin concentration; higher SOD, APX and CAT activities; higher photosynthetic rate and chlorophyll content index; lower H_2_O_2_ concentration	Li et al. (2016)
Drought priming	Spring wheat	Heat stress	Higher ABA concentration; lower RWC and transpiration rate; higher A_sat_^a^ and V_max_^b^	Wang et al. (2015)
Drought priming	Wheat	Heat stress	Higher leaf water potential and chlorophyll content; higher carbon assimilation and agronomic nitrogen-use efficiency; higher grain yield	Liu et al. (2017)
Drought priming (parent plants)	Winter wheat	High temperature stress (offspring)	Higher SOD and POD activities; lower MDA and H_2_O_2_ content; accumulation of heat shock proteins and up-regulation of sucrose synthesis	Zhang et al. (2016)
Drought stress	Tall fescue	Heat tolerance	Higher amount of phospholipids, glycolipids, phosphatidic acid, phosphatidylcholine, phosphatidylinositol, phosphatidylglycerol, and digalactosyl diacylglycerol; higher RWC^c^, chlorophyll content, photochemical efficiency	Zhang et al. (2019a
Drought priming	Tall fescue and Arabidopsis	Heat tolerance	Up-regulation of *CDPK3*, *MPK3*, *DREB2A*, *AREB3*, *MYB2*, *MYC4*, *HsfA2*, *HSP18*, and *HSP70*	Zhang et al. (2019b)
Drought treatment	Olive	Heat and UV-B radiation shock	Lower cell membrane permeability and water loss; Mitigating effect on quantum yield of PSII	Silva et al. (2018)

^a^*A*_*sat*_ saturated net photosynthesis rate

^b^*V*_*max*_ the maximum carboxylation rate of Rubisco

^c^*RWC* relative water content

### Cross-stress tolerance from drought to cold

Research has shown that a primary exposure to drought could affect a second stress also in form of cold or frost (Table 1). For example, drought stress plays a dominant role by inducing cold tolerance in strawberry (*Fragaria* × *ananassa*) plants (Rajashekar and Panda 2014). The Mediterranean *Pinus nigra* exposed to an extreme drought stress acquired tolerance to low temperatures occurring in the following years (Kreyling et al. 2012). Li et al. (2015) found that wheat plants pre-exposed to moderate drought stress at the vegetative stage had improved cold tolerance at the stem elongation stage by sustaining ROS homeostasis, reducing leaf water loss, decreasing oxidative injuries of the photosynthetic apparatus, and increased ABA levels. In barley plants, drought priming could also induce cold tolerance, which could be enhanced by application of exogenous melatonin (Li et al. 2016). Studies on three different genotypes of *Coffea* spp. showed that water deficit during the cold season was beneficial to alleviate the effect of cold stress by increased anti-oxidative defense (Ramalho et al. 2018). These examples confirm that drought-primed plants possess higher RWC and ABA levels, more active antioxidant defense systems, and higher chlorophyll contents and photosynthetic rates than non-primed plants under low temperature stress. The improved protection results in higher grain yield and increased tolerance compared to non-primed plants.

### Cross-stress tolerance from drought to heat

Heat stress limits the growth and productivity of temperate plant species, therefore heat tolerance is needed for growth in areas with high temperature. Drought priming as a suitable method for improving heat tolerance has been reported for various plant species (Table 1). Research with spring wheat (*Triticum aestivum* L. cv. Vinjett) showed that pre-exposure to drought stress during stem elongation improved tolerance to high temperature occurring later during grain filling (Wang et al. 2015). The effects of drought priming on grain yield during early developmental stages and nitrogen-use efficiency have been studied in wheat plants under post-anthesis heat stress. Moderate drought stress at the 5th-leaf stage of wheat plants improved carbon assimilation and agronomic nitrogen-use efficiency during later heat stress, resulting in higher grain yield and enhanced stress tolerance (Liu et al. 2017). Zhang et al. (2016) reported that drought priming executed on parent plants could stimulate a cross tolerance in their offspring in winter wheat plants under heat stress conditions. The effect of cross-stress tolerance on lipidomic profiles was analysed in tall fescue (*Festuca arundinacea*) by Zhang et al. (2019a). The results suggest that drought-primed plants enhanced tolerance to a subsequent heat stress through reprogramming of lipid metabolism and stress signaling (Zhang et al. 2019a). Foliar application of ABA has also a positive role in drought priming-enhanced heat tolerance in tall fescue and *A. thaliana*, which is associated with the transcriptional up-regulation of genes related to heat protection, ABA responses and stress signaling (Zhang et al. 2019b). The phenomenon of cross-stress tolerance was also reported for olive plants (Silva et al. 2018). Olive plants grown under non-irrigated conditions could prevent cumulative damages by heat and UV-B radiation by modulating some tolerance mechanisms compared to well-irrigated plants (Silva et al. 2018). The underlying mechanisms refer to the physiological processes, metabolic pathways, and the regulatory network of genes, contributing to drought priming-improved heat tolerance.

Another type of cross-stress tolerance was reported by Herms and Mattson (1992), they showed that previous exposure to abiotic stress (e.g. drought stress) could induce herbivore resistance by increasing carbon-based secondary metabolites. There are many examples that cross memory takes place between drought and cold stress, between drought and heat stress, or even between biotic stress and abiotic stress in different plant species, including *A. thaliana*, strawberry, *P. nigra*, spring wheat, etc*.* (Shinozaki and Yamaguchi-Shinozaki 2000; Kreyling et al. 2012; Rajashekar and Panda 2014; Li et al. 2015; Wang et al. 2015). Cross-stress tolerance is regulated by a complex network involving the interaction of multiple external and internal factors (Munné-Bosch and Alegre 2013). Due to the irregular and variable growth environments, cross-stress tolerance is extremely important in overcoming unpredictable and diverse stresses and more attention should be paid to it in agricultural practices.

## Priming of seeds

Seed priming has been developed as a low-cost and efficient approach to increase crop yield and to increase tolerance against various stresses (Jisha et al. 2013; Sher et al. 2019). According to the difference of priming agents, seed priming is classified into different types (Sher et al. 2019). Seed priming is not only promoting seed germination and improving plant growth and crop yield, but it also increases tolerance against abiotic stress under changing environmental conditions (Sher et al. 2019; Marthandan et al. 2020). Examples for seed priming which lead to drought tolerance are briefly described in Table 2. The current review focuses on seed priming correlated to drought tolerance.

**Table 2:** Examples of seed priming-induced drought tolerance in plants

Types	Chemicals	Species	Responsible factors	References
Hydropriming	Water	Maize	Significantly improved germination index, seedling vigour index and length of seedling	Janmohammadi et al. (2008)
Hydropriming and osmopriming	Water and mannitol (4%)	Chickpea	Higher activities of amylase, invertases (acid and alkaline), sucrose synthase and sucrose phosphate synthase; longer root and shoot length	Kaur et al. (2002)
Hydropriming and osmopriming	Water and PEG^a^	Rice	Higher proline and soluble protein content, PAL^b^, SOD, CAT, and POD activities; lower soluble sugar and MDA content; accelerated glucose metabolism	Sun et al. (2010)
Hydropriming and osmopriming	Water and PEG-6000	*Cleistogenes songorica*	Lower lipid peroxidation and H_2_O_2_ content; greater antioxidant enzyme activity; higher nuclear DNA contents during cell cycle	Tao et al. (2018)
Hydropriming and osmopriming	Water and CaCl_2_	Cotton	Higher emergence index, mean germination time, number of bolls per plant, boll weight per plant, lint weight, seed weight, plant height	Nasir et al. (2019)
Osmopriming	CaCl_2_ (− 1.25 MPa)	Wheat	Improved leaf area index, leaf area duration, and crop growth rate	Hussain et al. ((2018)
Osmopriming	Melatonin	Rapeseed	Improved stomatal number, length, width, and cell wall strength; higher antioxidant system activities	Khan et al. (2019)
Biopriming	Mycorrhiza fungi	Sesame	Higher amount of chlorophyll index, nitrogen, phosphorus, potassium, zinc, iron and copper uptake; Lower water consumption	Askari et al. (2018)
Biopriming	*Pseudomonas fluorescens*	Okra	Higher RWC, sugar, and free amino acids content; higher activity of phenolics, ascorbate glutathione, SOD, CAT, APX and GPX; alleviated membrane damage and protein denaturation	Pravisya et al. (2019)
Solid matrix priming	Multi-walled carbon nanotubes	Caucasian alder	Higher seed vigour index, root and stem lengths, and dry weights	Rahimi et al. (2016)
Solid matrix priming	Multi-walled carbon nanotubes	Hopbush	Improved seed germination percentage, mean germination time, root and stem lengths, fresh and dry weights of root and stem	Yousefi et al. (2017)
Nutripriming	Zinc (ZnSO_4_)	Durum wheat	Higher seedling height and SOD activity; better seed germination	Candan et al. (2018)
Nutripriming	Mg (NO_3_)_2_ and ZnSO_4_	Wheat	Higher yield and yield attributes parameters (spike length, spike number, spike weight, seed number)	Singhal et al. (2019)
Nutripriming	Zinc (ZnSO_4_)	Wheat	Higher dissipation of excess energy; higher leaf succulence values	Pavia et al. (2019)
Hormonal priming	Auxin, cytokinin, gibberellin, cytokinin, ABA	Tall wheatgrass	Higher CAT, GR, POD, SOD activities; higher germination percentage and rate of germination	Eisvand et al. (2010)
Hormonal priming	Gibberellic acid	Wheat	Better growth and development; higher yield	Ulfat et al. (2017)
Hormonal priming	Auxin	Wheat	Higher grain yield	Bagheri et al. (2019)

^a^*PEG* polyethylene glycol

^b^*PAL* phenylalanine ammonia lyase

Hydropriming of maize (*Zea mays* L.) significantly improved germination as well as seedling growth under drought stress conditions (Janmohammadi et al. 2008). Hydroprimed cotton seeds (*Gossypium hirsutum* L.) obtained better germination parameters, growth and higher yield under water shortage conditions (Nasir et al. 2019). In the grass *Cleistogenes songorica* native to northern China, hydropriming treatments alleviated the detrimental effects of drought stress by decreasing lipid peroxidation and ROS accumulation, and increasing activities of antioxidant enzymes (Tao et al. 2018).

In comparison to seedlings from non-primed chickpea seeds, seedlings obtained from seeds primed with mannitol (4% w/v) had longer roots and shoots under water deficit conditions (Kaur et al. 2002). Primed (treated with different concentrations of PEG) and non-primed seeds of four rice cultivars were germinated under drought stress (imitated with PEG) to examine the effects of seed priming and physiological characteristics of rice plants (Sun et al. 2010). The study showed that a suitable concentration of PEG improved germination indices, as well as quality and drought tolerance of seedlings under drought stress. The physiological changes were correlated with an increase in proline, soluble proteins, phenylalanine ammonia lyase (PAL), peroxidase (POD), SOD and CAT, and decreased soluble sugars and MDA (Sun et al. 2010).The effect of seed osmopriming (with CaCl_2_ solution) on wheat yield was evaluated in a field experiment (Hussain et al. 2018). The osmoprimed seeds led to increased yield and crop allometry and improved productivity under drought stress, due to establishment of early and uniform tolerance mechanisms (Hussain et al. 2018). Melatonin-primed rapeseeds had better germination parameters and subsequent better seedling growth under drought stress. This phenomenon is ascribed to the effects of melatonin-priming, including improvement of stomatal traits, strengthening of cell walls, enhanced activities of enzymatic and non-enzymatic antioxidants, and accumulation of osmo-protectants (Khan et al. 2019).

Seed priming using 50 ppm of auxin increased seed germination and the number of seminal roots whereas 100 ppm of gibberellin, 50 ppm of cytokinin, and 50 ppm of ABA improved seed performance under drought stress conditions, respectively (Eisvand et al. 2010). Research on wheat plants showed that hormonal priming with gibberellic acid or auxin significantly enhanced the growth and development of wheat plants, resulting in higher grain yield compared with non-primed plants (Ulfat et al. 2017; Bagheri et al. 2019).

These and related observations could contribute to the development of drought tolerant crop plants. In general, the overall growth of plants is enhanced under drought stress treatments. This could be further explored in future studies of plant priming or plant stress memory.

## Priming of resurrection plants

Not only seeds but also desiccation tolerant resurrection plants are responsive to priming and subsequently a stress memory is build-up. Stress memory was observed in the resurrection plant *Craterostigma plantagineum* which belongs to the *Linderniaceae* family. After being exposed to four dehydration/rehydration treatment cycles, expression of four representative stress-related genes and ROS pathway-related genes gradually increased, accompanied by increasing levels of SOD activity, proline content and sucrose content, conversely the H_2_O_2_ content and electrolyte leakage (EL) decreased, which indicates a gain of stress tolerance and also points to a stress memory (Liu et al. 2019; Liu 2020). Similarly, the resurrection plant *B. hygrometrica* acquired desiccation tolerance after priming through a pretreatment of slow dehydration (Zhu et al. 2015). Mitra et al. (2013) proposed that histone modifications in *B. hygrometrica* are altered during drought acclimation and are retained, which causes activation of downstream genes during subsequent desiccation. Metabolomes were compared between the acclimated and non-acclimated desiccation tolerant plant *Myrothamnus flabellifolia* Welw. (Bentley and Farrant 2020), and it was shown that long-term acclimation results in large-scale reprogramming of the metabolome (Bentley and Farrant 2020).

Cross-stress tolerance between desiccation and freezing temperatures was found in resurrection plants like *Haberlea rhodopensis* Friv. and *Ramonda myconi*. Georgieva et al. (2021) reported that *H. rhodopensis* Friv. has unique properties and can withstand desiccation as well as freezing temperatures. The adaptation of *H. rhodopensis* to low temperature is based on the readjustment of the photosynthetic apparatus, which has undergone modifications during primary desiccation (Mihailova et al. 2020). The resurrection plant *R. myconi* has been used to investigate the physiological mechanisms underpinning cross-stress tolerance between desiccation and freezing (Fernández-Marín et al. 2020). The results showed that protection of chloroplast structures are a response common for desiccation and low temperature in *R. myconi* (Fernández-Marín et al. 2020).

Common responses are observed during seed priming and priming of resurrection plants. The re-synthesis of degraded proteins upon rehydration based on stable storage of transcripts in the desiccated state, the increase of tocopherol in membranes upon desiccation, and the expression of targeted genes and proteins during dehydration are common dehydration responses in seeds and vegetative tissues (Oliver et al. 2020). Despite these observations, it is not possible at present to firmly conclude that the priming mechanisms are identical in seeds and resurrection plants.

It stimulated us to address the question whether the desiccation tolerant resurrection plant *C. plantagineum* could gain tolerance to biotic and/or abiotic stress, through similar treatments as seed priming. Desiccated *C. plantagineum* plants can be treated with hydropriming, osmopriming, solid matrix priming, biopriming, nutripriming, hormonal priming, and thermopriming methods which have already been used for seed priming. If untreated *C. plantagineum* plants are treated in different ways, then stress memory responses are induced during a second treatment which is an example for cross-stress tolerance. However, if the experiments are done with already desiccated *C. plantagineum* plants, it may induce different responses compared to untreated plants. This means that the physiological condition of the plant which undergoes priming treatments is important for the response. This hypothesis might contribute to uncover a new sight of plant priming or plant stress memory.

## Concluding remarks and future perspectives

**Fig. 1:**
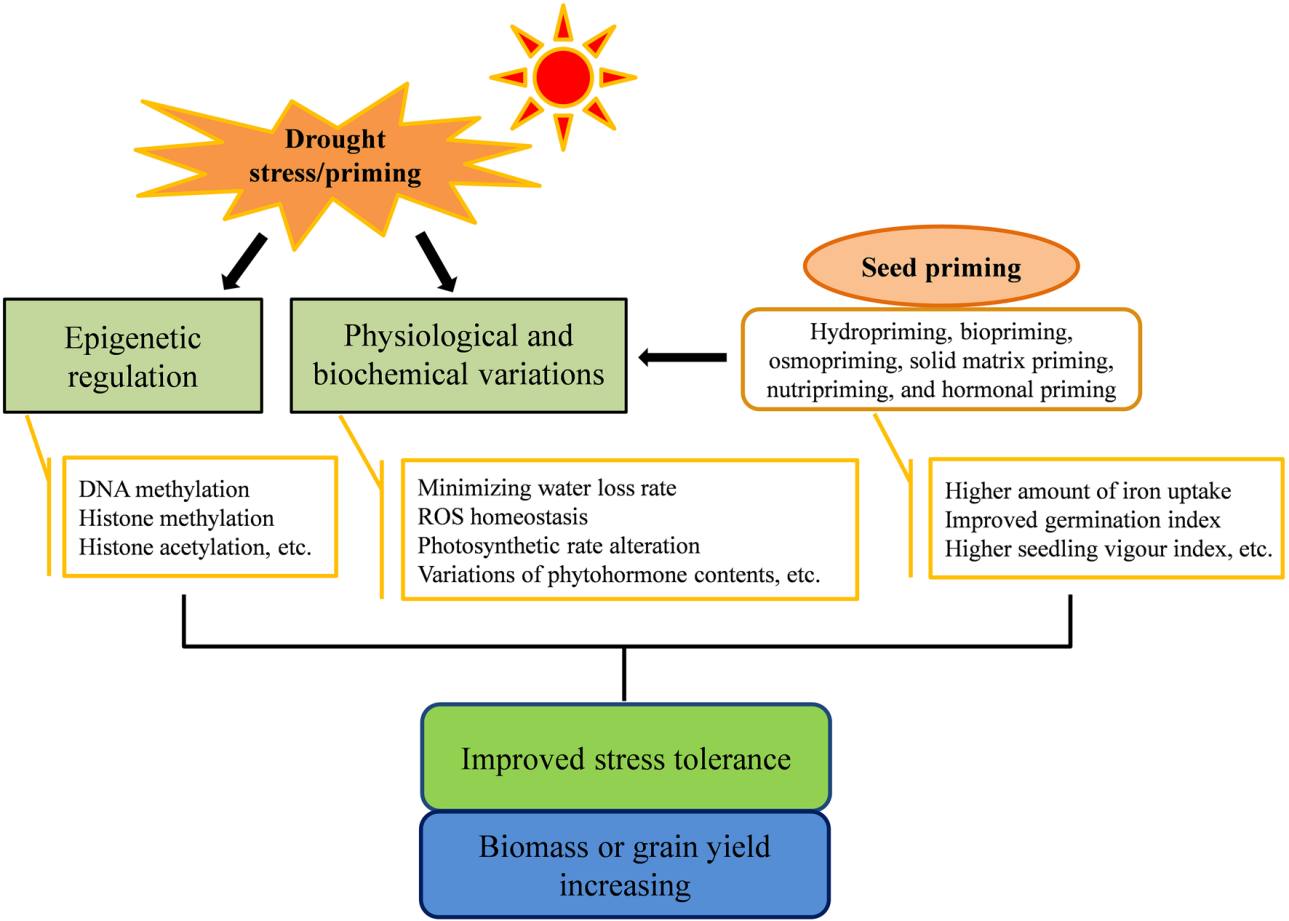
Overview of drought stress responses and seed priming in the context of drought tolerance in plants

Climate change and unevenly distributed precipitations seriously affect global agricultural production and food security. How to minimize adverse effects of climate change and to meet the food demand of the increasing human population becomes an urgent problem. Considering the conventional techniques (e.g. selection and hybridization) and recently genetic engineering (e.g. transgenic technology, gene mutation and polyploidy breeding) have limitations, such as the length of time, large man power, or the restrictions based on biosafety issues and environmental protection problems. Hence, alternative technologies emerged as promising solutions. As we describe in this review, methods involved in plant stress memory, cross-stress tolerance, and seed priming have become effective and favorable techniques for environmentally friendly and sustainable agriculture (Fig. 1). Currently, there is increasing evidence in the literature how to alleviate the negative effects of dehydration stress based on the plant immune system. However, biochemical and molecular mechanisms of plant memory and seed priming still need to be investigated for precise and reliable applications of this approach. Future research needs to focus on the elucidation of how long the stress memory can persist, how to prolong and increase the positive effects of plant memory, and how to apply priming on a large scale to diverse plants.

## *Author contribution statement*

DB and XL conceived the manuscript. XL and WQ wrote the manuscript. DB, XL and WQ revised the article.
